# Survival and biomarker analysis for ovarian mucinous carcinoma according to invasive patterns: retrospective analysis and review literature

**DOI:** 10.1186/s13048-021-00783-3

**Published:** 2021-02-14

**Authors:** Taira Hada, Morikazu Miyamoto, Hiroki Ishibashi, Hiroko Matsuura, Takahiro Sakamoto, Soichiro Kakimoto, Hideki Iwahashi, Hitoshi Tsuda, Masashi Takano

**Affiliations:** 1 Department of Obstetrics and Gynecology, National Defense Medical College Hospital, 3-2, Namiki, Saitama 359-8513 Tokorozawa, Japan; 2 Department of Pathology, National Defense Medical College Hospital, 359-8513 Tokorozawa, Saitama Japan

**Keywords:** Ovarian mucinous carcinoma, Invasive pattern, Infiltrative invasion, Expansile invasion, 2020 World Health Organization, Prognosis

## Abstract

**Background:**

In ovarian mucinous carcinoma, invasive pattern is the important factor but there were less reposts to investigate it. The aim of this study was to examine the association between prognosis and invasive patterns of ovarian mucinous carcinoma and to investigate the biomarkers of the diagnosis and prognosis immunochemically. Patients with ovarian mucinous carcinoma at our institution between 1984 and 2018 were identified. A pathological review was conducted using the 2020 World Health Organization criteria. The prognosis of infiltrative invasion and expansile invasion of ovarian mucinous carcinoma were retrospectively compared. In addition, immunohistochemical staining was conducted for all cases, and the immunohistochemical differences between the two invasive patterns were compared.

**Results:**

After the pathological review, 25 cases with infiltrative invasion and 24 cases with expansile invasion were included. Ovarian mucinous carcinoma with infiltrative invasion showed significantly worse progression-free survival (PFS, *p* < 0.01) and overall survival (OS, *p* < 0.01) than those with expansile invasion. Multivariate analysis demonstrated that the pattern of infiltrative invasion was a worse prognostic factor for PFS (hazard ratio 9.01, *p* < 0.01) and OS (hazard ratio 17.56, *p* < 0.01). Immunohistochemically, cytokeratin (CK) 5/6 (*p* = 0.01), cluster of differentiation (CD) 24 (*p* = 0.02), and epithelial growth factor receptor (EGFR) (*p* < 0.01) were statistically related to infiltrative invasion. The PFS (*p* = 0.04) and OS (*p* = 0.02) of cases with EGFR-positive OMC were worse than those with negative OMC.

**Conclusions:**

Infiltrative invasion was observed to be a prognostic factor showing worse outcomes for ovarian mucinous carcinoma compared to expansile infiltration. CK5/6, CD24, and EGFR might be biomarkers of the diagnosis.

## Background

Ovarian carcinoma (OC) is the most lethal gynecological carcinoma [1]. The histology of OC is an important factor related to the prognosis, in addition to the International Federation of Gynecology and Obstetrics (FIGO) stage and residual tumor at surgery [2–7]. Histologically, OCs are classified into type I and type II based on distinctive morphological and molecular genetic features [8, 9]. Type I tumors are discovered at an early stage and have an indolent clinical course [8, 9]. In contrast, type II tumors are detected at an advanced stage and exhibit highly aggressive behavior [8, 9]. Because ovarian mucinous carcinoma (OMC) is classified as a type I tumor, it is considered a nonaggressive histological subtype.

The incidence of OMC among OCs ranges from 3 to 11 % [10]. The majority (65–80 %) of OMC cases are diagnosed at an early stage [11]. OMC is known to be poorly responsive to conventional chemotherapy, and its prognosis depends on the FIGO stage and residual tumor size after the primary surgery [4–7, 11–13]. The prognosis of OMC in the early stage is not different from that of other histological subtypes; however, it is worse in the advanced stage [10, 11]. Thus, a new treatment for OMC is needed.

According to the 2020 World Health Organization (WHO) classification, the invasive patterns of OMC are classified into two categories: infiltrative invasion and expansile invasion [14]. Infiltrative invasion is characterized by irregular glands, nests, and single cells with malignant cytological features infiltrating the stroma, which is often desmoplastic. Expansile invasion is characterized by marked glandular crowding with little intervening stroma, creating a labyrinthine appearance, and a cribriform pattern might be present. In addition to the above-mentioned prognostic factors, several reports have demonstrated that the invasive pattern is associated with the prognosis of OMC [15–20]. We considered that a study examining the relationship between the invasive patterns and prognosis of OMC is necessary owing to the rarity of OMC.

The aim of the present study was to revalidate the differences in the clinicopathological features, prognosis, and immunohistochemical features between infiltrative invasion and expansile invasion of OMC through a pathological review. Further, we reviewed the existing literature.

## Results

**Fig. 1:**
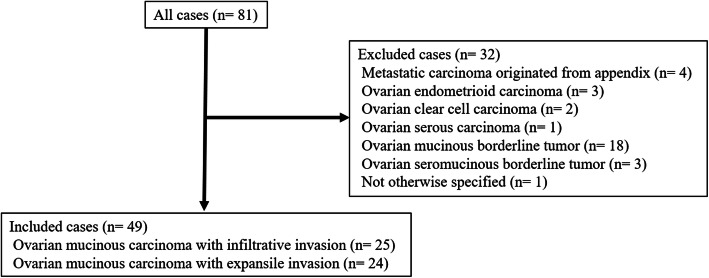
Change of histological type in all cases after a pathological review Eighty-one cases of ovarian mucinous carcinoma (OMC) were initially identified. After a pathological review, 4 cases were diagnosed with metastatic carcinoma originating from the appendix, 3 cases were diagnosed with ovarian endometrioid carcinoma, 2 cases with ovarian clear cell carcinoma, 1 case with ovarian serous carcinoma, 18 cases with ovarian mucinous borderline tumor, 3 cases with ovarian seromucinous borderline tumor, and 1 case was not otherwise specified; additionally, these cases were excluded. Finally, 25 cases of OMC with infiltrative invasion and 24 cases of OMC with expansile invasion were included

A total of 81 patients with OMC were identified. The results of the pathological review are shown in Fig. 1. The pathological diagnosis of 49 cases was OMC and that of 32 cases was changed from OMC to other histological subtypes, including metastatic carcinoma originating from the appendix in 4 cases, ovarian endometrioid carcinoma in 3 cases, ovarian clear cell carcinoma in 2 cases, ovarian serous carcinoma in 1 case, ovarian mucinous borderline tumor in 18 cases, ovarian seromucinous borderline tumor in 3 cases, and not otherwise specified in 1 case.

**Fig. 2:**
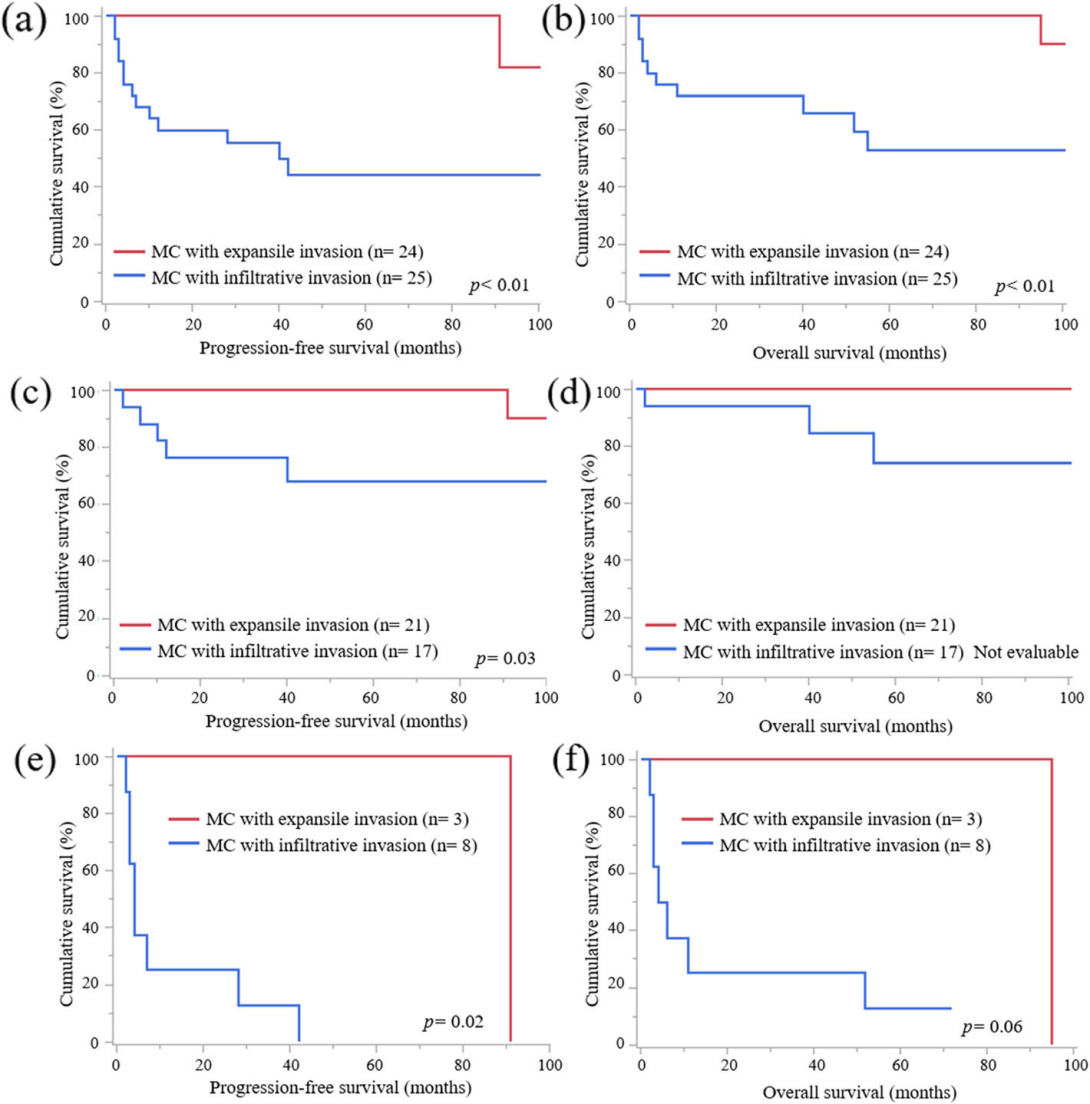
PFS and OS curves according to FIGO stage status **a** PFS curves of cases of OMC with infiltrative invasion and expansile invasion in all FIGO stages. OMC with infiltrative invasion showed worse PFS than OMC with expansile invasion. **b** OS curves of cases of OMC with infiltrative invasion and expansile invasion in all FIGO stages. OMC with infiltrative invasion exhibited worse OS than OMC with expansile invasion. **c** PFS curves of cases of OMC with infiltrative invasion and expansile invasion in FIGO stage I and II. OMC with infiltrative invasion showed worse PFS than OMC with expansile invasion. **d** OS curves of cases of OMC with infiltrative invasion and expansile invasion in FIGO stage I and II. There were no statistical significances about OS between two groups. **e** PFS curves of cases of OMC with infiltrative invasion and expansile invasion in FIGO stage III and IV. OMC with infiltrative invasion showed worse PFS than OMC with expansile invasion. **d** OS curves of cases of OMC with infiltrative invasion and expansile invasion in FIGO stage III and IV. There were no statistical significances about OS between two groups

Among the 49 patients diagnosed with OMC, 25 (51 %) cases with infiltrative invasion and 24 (49 %) cases with expansile invasion were included in our analysis. The median observation period in the OMC cases was 53 (1–262) months. The characteristics of OMC according to the invasive patterns are listed in Table 1. It was observed that OMC with infiltrative invasion was more frequently treated with adjuvant chemotherapy (*p* = 0.02) and more often recurred (*p* < 0.01) than OMC with expansile invasion. There was no statistical difference in the response rate between the two groups; however, all cases with expansile invasion that had residual tumor at primary debulking surgery (PDS) achieved complete response or partial response after adjuvant chemotherapy. Other factors showed no statistically significant differences between the two groups. OMC with infiltrative invasion showed worse PFS (Fig. 2a, *p* < 0.01) and OS (Fig. 2b, *p* < 0.01) than OMC with expansile invasion in all FIGO stages. In FIGO stage I and II, OMC with infiltrative invasion showed worse PFS than OMC with expansile invasion (Fig. 2c, *p* = 0.03), but OS was not evaluable because no patients with expansile invasion died for diseases (Fig. 2d). Also, in FIGO stage III and IV, OMC with infiltrative invasion showed worse PFS (Fig. 2e, *p* = 0.02) but not OS than OMC with expansile invasion (Fig. 2f , *p* = 0.06).

**Table 1:** Characteristics of 49 patients with ovarian mucinous carcinoma according to invasive patterns

	Ovarian mucinous carcinoma with infiltrative invasion	Ovarian mucinous carcinoma with expansile invasion	*p*-Value
Variables	*n* = 25	*n* = 24	
Age (years)			0.16
< 51	16 (64)	10 (42)	
≥ 51	9 (36)	14 (58)	
FIGO stage (%)			0.26
I	16 (64)	20 (84)	
II	1 (4)	1 (4)	
III	6 (24)	1 (4)	
IV	2 (8)	2 (8)	
Tumor site (%)			0.35
Unilateral	21 (84)	23 (96)	
Bilateral	4 (4)	1 (4)	
Standard surgery (%)			0.78
Yes	15 (60)	13 (54)	
No	10 (40)	11 (46)	
Peritoneal cytology (%)			0.78
Positive	12 (48)	10 (42)	
Negative	13 (52)	14 (58)	
Residual tumor diameter at PDS (%)			0.58
No residual tumor	18 (72)	20 (83)	
< 1.0 cm	1 (4)	1 (4)	
≥ 1.0 cm	6 (24)	3 (13)	
Adjuvant chemotherapy (%)			0.02
Not done	4 (16)	12 (50)	
Done	21 (84)	12 (50)	
Regimen of the adjuvant chemotherapy			
Paclitaxel and carboplatin	5 (24)	2 (17)	0.20
Platinum-based therapy ^a^	16 (76)	10 (83)	
Response rate of adjuvant chemotherapy for patients with residual tumor which received adjuvant chemotherapy (%)			0.20
CR/PR	3 (43)	3 (100)	
S D/PD	4 (57)	0 (0)	
Recurrence (%)			< 0.01
Yes	13 (52)	2 (8)	
No	12 (48)	22 (92)	

Abbreviations*:SD* standard deviation; *FIGO* International Federation of Gynecology and Obstetrics; *PDS* primary debulking surgery; *cm* centimeter; *CR* complete response; *PR* partial response; *SD* stable disease; *PD* progressive disease

^a^ Platinum-based therapy included the combination with cyclophosphamide, doxorubicin, and cisplatin, the combination with cyclophosphamide and cisplatin, the combination with etoposide and cisplatin, and the combination with irinotecan and nedaplatin

The univariate analysis of PFS and OS revealed that FIGO stage, residual tumor at PDS, and invasive pattern were prognostic factors. The multivariate analysis for PFS and OS using these variables revealed that infiltrative invasion was a worse prognostic factor for PFS (hazard ratio [HR] 9.01, *p* < 0.01) and OS (HR 17.56, *p* < 0.01) (Table 2).

**Table 2:** Univariate and multivariate analysis for PFS and OS in all patients with OMC

	Progression-free survival				Overall survival			
	Univariate analysis		Multivariate analysis		Univariate analysis		Multivariate analysis	
Variables	HR (95 % CI)	*p*-value	HR (95 % CI)	*p*-value	HR (95 % CI)	*p*-value	HR (95 % CI)	*p*-value
Age (years)								
≥51 vs. <51	0.51 (0.17–1.42)	0.20			0.71 (0.20–2.35)	0.57		
FIGO stage								
I vs. II, III, IV	0.11 (0.04–0.32)	< 0.01	0.12 (0.02–0.61)	0.01	0.06 (0.01–0.22)	< 0.01	0.09 (0.01–0.84)	0.04
Residual tumor at PDS								
Optimal vs. Suboptimal	0.17 (0.06–0.50)	< 0.01	0.95 (0.20–3.55)	0.94	0.09 (0.02–0.29)	< 0.01	0.37 (0.05–1.68)	0.21
Invasive pattern								
Infiltrative invasion vs. Expansile invasion	8.69 (2.37–55.86)	< 0.01	9.01 (2.28–61.41)	< 0.01	12.52 (2.37-230.59)	< 0.01	17.56 (2.58-393.24)	< 0.01

Abbreviations: *PFS* Progression-free survival, *OS* Overall survival, *OMC* ovarian mucinous carcinoma, *HR* hazard ratio, *CI* confidence interval, *PDS* primary debulking surgery

**Fig. 3:**
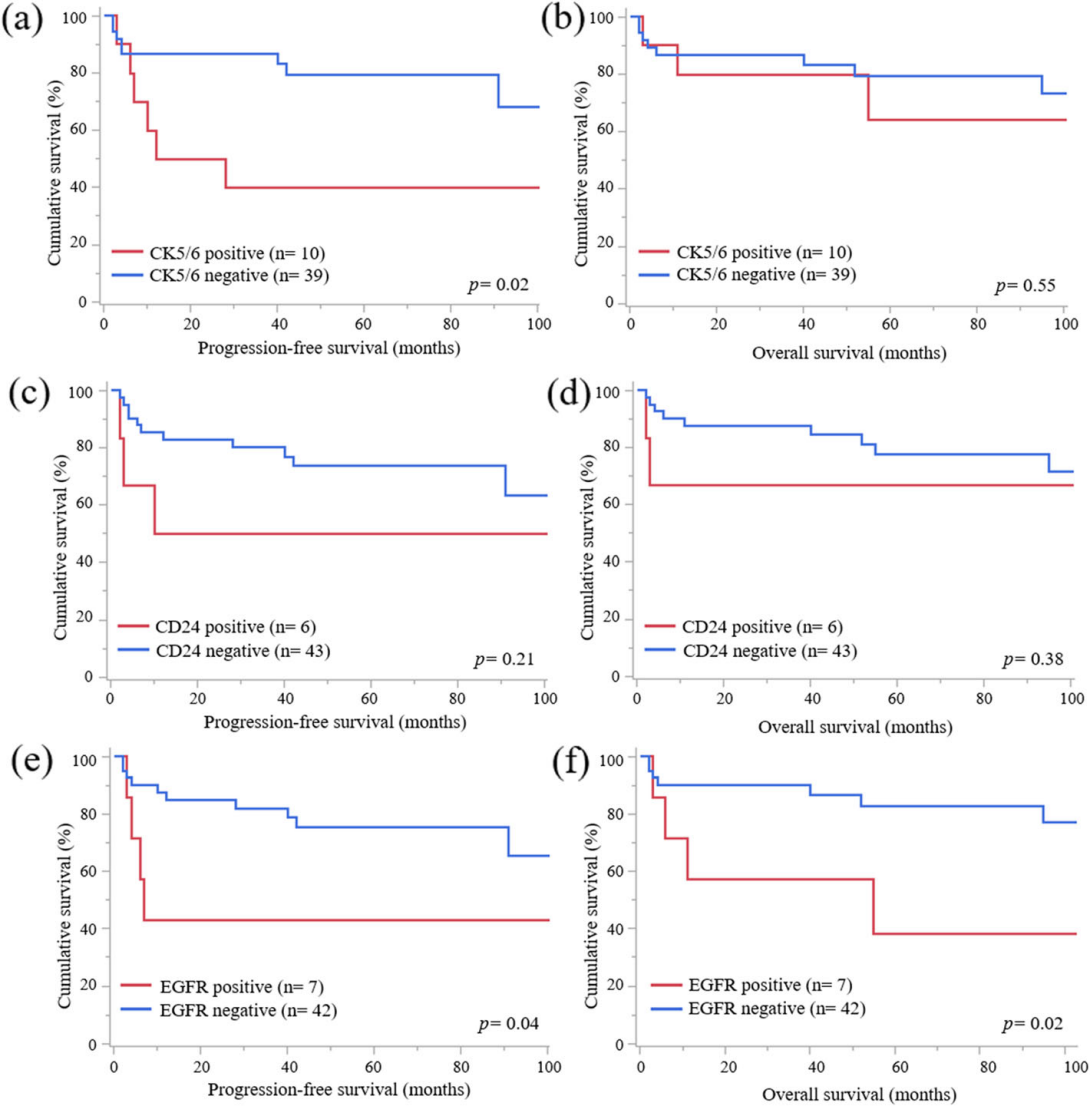
PFS and OS curves according to CK5/6, CD24, and EGFR status **a** PFS curves of cases according to the CK5/6 status. Cases which were positive for CK5/6 had significantly worse prognosis about PFS. **b** OS curves of cases according to the CK5/6 status. There was no statistical significance between 2 groups. **c** PFS curves of cases according to the CD24 status. There was no statistical significance between 2 groups. **d** OS curves of cases according to the CD24 status. There was no statistical significance between 2 groups. **e** PFS curves of cases according to the EGFR status. Cases which were positive for EGFR had significantly worse prognosis about PFS. **f** OS curves of cases according to the EGFR status. Cases which were positive for EGFR had significantly worse prognosis about OS

The results of immunohistochemical staining for each invasive pattern are listed in Table 3. It is notable that more cases of OMC with infiltrative invasion were positive for cytokeratin 5/6 (CK5/6) (*p* = 0.01), cluster of differentiation 24 (CD24) (*p* = 0.02), and epithelial growth factor receptor (EGFR) (*p* < 0.01) than those of OMC with expansile invasion. Only two cases were positive for all CK5/6, CD24, and EGFR. There were no statistical differences between several proteins and invasive patterns. The patients with CK5/6-positive OMC had worse PFS (Fig. 3a, *p* = 0.02) but not OS (Fig. 3b, *p* = 0.55) than those with negative OMC. There were no statistical differences of PFS (Fig. 3c, *p* = 0.21) and OS (Fig. 3d, *p* = 0.38) according to CD24 status. The patients with EGFR-positive OMC had worse PFS (*p* = 0.04) and OS (*p* = 0.02) than those with negative OMC (Fig. 3e and f). There were no statistical significances of PFS (*p* = 0.53) and OS (*p* = 0.36) between patients with or without positive CK5/6, CD24, and EGFR. However, there were no statistically significant differences between other proteins and the prognosis.

**Table 3:** The results of immunohistochemistry staining for ovarian mucinous carcinoma

		Ovarian mucinous carcinoma with infiltrative invasion		Ovarian mucinous carcinoma with expansile invasion		*p*-Value
Molecule		*n* = 25		*n* = 24		
CK7 (n (%))	Positive	24	(96.0)	24	(100.0)	0.99
	Negative	1	(4.0)	0	(0.0)	
CK20 (n (%))	Positive	11	(44.0)	15	(62.5)	0.24
	Negative	14	(56.0)	9	(37.5)	
CDX2 (n (%))	Positive	21	(84.0)	23	(95.8)	0.35
	Negative	4	(16.0)	1	(4.2)	
HER-2 (n (%))	Positive	7	(28.0)	10	(41.7)	0.38
	Negative	18	(72.0)	14	(58.3)	
CK 5/6 (n (%))	Positive	9	(36.0)	1	(4.2)	0.01
	Negative	16	(64.0)	23	(95.8)	
Androgen receptor (n (%))	Positive	2	(8.0)	0	(0.0)	0.49
	Negative	23	(92.0)	24	(100.0)	
ALDH1 (n (%))	Positive	9	(36.0)	10	(41.7)	0.77
	Negative	16	(64.0)	14	(58.3)	
CD24 (n (%))	Positive	6	(24.0)	0	(0.0)	0.02
	Negative	19	(76.0)	24	(100.0)	
CD133 (n (%))	Positive	8	(32.0)	5	(20.8)	0.52
	Negative	17	(68.0)	19	(79.2)	
PD-1 (n (%))	Positive	2	(8.0)	0	(0.0)	0.49
	Negative	23	(92.0)	24	(100.0)	
PD-L1 (n (%))	Positive	6	(24.0)	6	(25.0)	0.99
	Negative	19	(76.0)	18	(75.0)	
ZEB1 (n (%))	Positive	1	(4.0)	1	(4.2)	0.99
	Negative	24	(96.0)	23	(95.8)	
c-Met (n (%))	Positive	6	(24.0)	4	(16.7)	0.73
	Negative	19	(76.0)	20	(83.3)	
EGFR (n (%))	Positive	7	(28.0)	0	(0.0)	< 0.01
	Negative	18	(72.0)	24	(100.0)	
Snail-2 (n (%))	Positive	1	(4.0)	1	(4.2)	0.99
	Negative	24	(96.0)	23	(95.8)	

Abbreviations: *CK7* Cytokeratin 7, *CK20* Cytokeratin 20, *CDX2* Caudal type homeobox 2, *HER-2* Human epidermal growth factor receptor 2, *CK5/6* Cytokeratin 5/6, *ALDH1* Aldehyde dehydrogenase 1, *CD24* Cluster of differentiation 24, *CD133* Cluster of differentiation 133, *PD-1* Programmed cell death 1, *PD-L1* Programmed cell death 1- ligand 1, *ZEB1* Zinc finger E-box binding homeobox 1, *EGFR* Epithelial growth factor receptor

## Discussion

In our study, 25 (51.0 %) cases with infiltrative invasion and 24 (49.0 %) cases with expansile invasion were included. The cases with infiltrative invasion were more frequently treated with adjuvant chemotherapy and more often showed recurrence. Furthermore, the PFS and OS of cases with infiltrative invasion were worse than those of cases with expansile invasion; multivariate analysis revealed that infiltrative invasion was a negative prognostic factor for PFS and OS. In addition, immunohistochemical staining revealed that CK5/6, CD24, and EGFR were related to infiltrative invasion compared with expansile invasion.

The findings of the review of literature reports published from 2000, including the present study, on the invasive patterns of OMC identified through a pathological review are shown in Table 4 [15–19]. According to our review, infiltrative invasion was relatively frequently diagnosed (135 of 250 cases, 54 %). Furthermore, the cases with an infiltrative invasion pattern were discovered at a more advanced stage (*p* < 0.01), had a higher recurrence rate (40 % vs. 7 %, *p* < 0.01), had a higher mortality rate (33 % vs. 9 %, *p* < 0.01), and showed worse prognoses than cases with expansile invasion. Thus, although previous reports identified OMC as type I OC and a nonaggressive tumor [8, 9], OMC with infiltrative invasion was considered to be an aggressive subtype.

**Table 4:** Findings of literature review on invasive patterns of ovarian mucinous carcinoma including the present study

Authors	Total no. of cases	No. of cases in each invasive pattern (%)		FIGO stage		*p*-Value	Recurrence (%)	*p*-Value	Prognosis		*p*-Value
				I	II–IV				Alive with and without diseases	Death due to diseases or any reason	
Lee and Scully [15]	21	Infiltrative invasion	11 (52)	5 (46)	6 (54)	0.01	6 (54)	0.01	5 (46)	6 (54)	0.01
		Expansile invasion	10 (48)	10 (100)	0 (0)		0 (0)		10 (100)	0 (0)	
Rodriguez and Prat [16]	26	Infiltrative invasion	15 (58)	9 (60)	6 (40)	0.02	9 (60)	< 0.01	7 (47)	8 (53)	0.04
		Expansile invasion	11 (42)	11 (100)	0 (0)		0 (0)		10 (91)	1 (9)	
Muyldermans et al. [17]	44	Infiltrative invasion	21 (48)	12 (57)	9 (43)	0.01	9 (43)	0.01	12 (57)	9 (43)	0.04
		Expansile invasion	23 (52)	21 (91)	2 (9)		2 (9)		20 (87)	3 (13)	
Khunamornpong et al. [18]	46	Infiltrative invasion	28 (61)	22 (79)	6 (21)	0.07	11 (39)	0.02	20 (71)	8 (29)	0.07
		Expansile invasion	18 (39)	18 (100)	0 (0)		1 (6)		17 (94)	1 (6)	
Gouy et al. [19]	64	Infiltrative invasion	35 (55)	35 (100)	-	-	6 (17)	0.49	31 (89)	4 (11)	0.37
		Expansile invasion	29 (45)	29 (100)	-		3 (10)		28 (97)	1 (3)	
Present study	49	Infiltrative invasion	25 (51)	16 (64)	9 (36)	0.2	13 (52)	< 0.01	15 (60)	10 (40)	0.11
		Expansile invasion	24 (49)	20 (83)	4 (17)		2 (8)		20 (87)	4 (13)	
Total	250	Infiltrative invasion	135 (54)	99 (73)	36 (27)	< 0.01	54 (40)	< 0.01	90 (67)	45 (33)	< 0.01
		Expansile invasion	115 (46)	109 (95)	6 (5)		8 (7)		105 (91)	10 (9)	

The diagnosis of OMC is cumbersome. The metastatic carcinomas from other sites are often misdiagnosed as primary OMC because of the histological resemblance between these tumors [10, 21]. In fact, out of the cases initially diagnosed as OMC, 50–70 % were diagnosed with metastatic carcinoma after a pathological review [10]. In our study, 4 of 81 (5 %) cases were diagnosed with metastatic carcinoma originating from the appendix. The number of metastatic carcinoma cases was lower in this study than in previous reports because we preoperatively performed gastroscopy and colonofiberscopy in addition to the detailed examination of the appendix by a digestive surgeon. Furthermore, ovarian borderline tumors are often misdiagnosed as primary OMC [22]. Previous reports show that OMC often exhibits a continuum of differentiation from benign epithelium to borderline malignancy to invasive carcinoma, and ovarian mucinous borderline tumors resemble invasive well-differentiated OMCs [22, 23]. In our study, the diagnosis of 18 of 81 (22 %) cases was changed from OMC to ovarian mucinous borderline tumors, which indicated the difficulty in the diagnosis. However, the association between CK5/6, CD24, and EGFR and invasive patterns was identified. These markers might be useful as biomarkers for diagnosis.

In a previous report, high-grade serous carcinoma was classified into four molecular subtypes, which were related to the prognosis [24]. A subsequent report showed the relationship between the histopathological classification and prognostically distinct gene expression subtypes of HGSC [25]. In the case of OMC, several gene mutations or amplifications have been reported [10]. *HER-2* amplification/overexpression and *KRAS* mutation in OMC were reported to be associated with a decreased likelihood of disease recurrence or death [26]. Furthermore, an association between *HER-2* expression and invasive patterns has been reported [27]. However, this relationship was not observed in our study.

CK5/6 was consistently expressed in the stratified epithelia and derived neoplasms, such as squamous cell carcinoma and was related to the prognosis of ovarian carcinoma [28, 29]. CD24 is one of the ovarian cancer stem cell markers, and the overexpression of CD24 in OCs was associated with a more aggressive phenotype and increased metastasis and invasion capacity [30–32]. EGFR is a growth-factor-receptor tyrosine kinase that is related to transformation of cellular phenotypes and growth and survival of tumor cells [33]. Additionally, EGFR was associated with poor overall and disease-free survival outcomes [34]. Our study showed that OMC with infiltrative invasion was more frequently positive for CK5/6, CD24, and EGFR, which might suggest that CK5/6, CD24, and EGFR were associated with the aggressive features of OMC with infiltrative invasion. Additionally, the EGFR status was associated with worse prognoses, and EGFR inhibitors might be a new strategy for OMC with infiltrative invasion.

In several previous report, the diagnosis of mucinous carcinoma was difficult because benign tumors, borderline tumors, and invasive carcinomas could coexist, and appropriate sampling of tissue was needed to diagnose small areas of invasion due to large size [23, 35–38]. In our study, OMC with infiltrative invasion was positive for CK5/6, CD24, and EGFR and these makers might be useful for the diagnostic biomarker.

This study had some limitations, including the small sample size from a single institution and retrospective analysis. As a result, our study included a small number of cases; however, our study had a large sample size relative to several other reports. Further large-scale studies are needed to confirm the clinical significance of OMC in the future.

## Conclusions

The invasive patterns were associated with prognoses, as previous reports have shown. Particularly, OMC with an infiltrative pattern showed worse prognoses and positive for CK5/6, CD24, and EGFR. Also, EGFR was related with the worse prognosis. These biomarkers may be useful for the development of new treatment strategy and diagnostic biomarker in the clinical practice.

## Methods

**Fig. 4:**
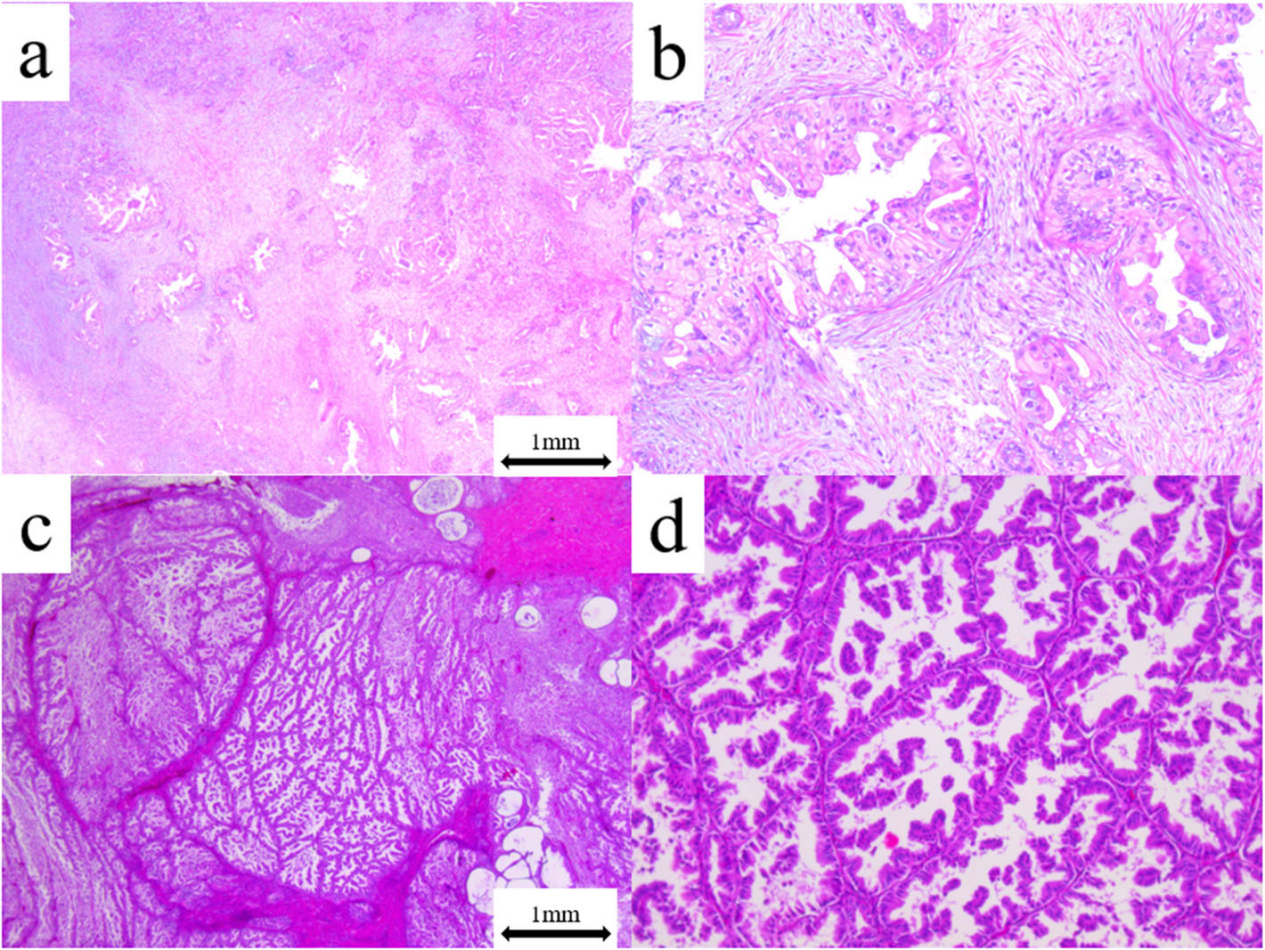
Representative images of ovarian mucinous carcinoma (OMC) with infiltrative invasion and expansile invasion **a** OMC with infiltrative invasion was defined as obvious evidence of destructive stromal invasion measuring > 5 mm in the greatest linear extent (x 20). **b** Stromal invasion was associated with malignant glands, cell nests, and a desmoplastic stromal reaction (x 100). **c** OMC with expansile invasion was defined as marked glandular crowding with little intervening stroma, creating a labyrinthine appearance with little intervening normal ovarian stroma (x 10). **d** A cribriform pattern was present (x 100)

Patients with OMC who underwent a minimum of unilateral salpingo-oophorectomy and resection of the primary lesion at our hospital between 1984 and 2018 were identified. Those without clinical information or surgical tissue were excluded. A pathological review was conducted using the 2020 WHO criteria [14], and cases with infiltrative invasion and those with expansile invasion were identified. Briefly, infiltrative invasion was defined as a destructive stromal invasion by malignant glands, cell nests, or individual cells with a desmoplastic stromal reaction. Expansile invasion was defined as a confluent glandular growth pattern with little intervention of normal ovarian stroma (minimal or no stromal invasion). The representative images of infiltrative invasion and expansile invasion of OMC are shown in Fig. 4.

**Fig. 5:**
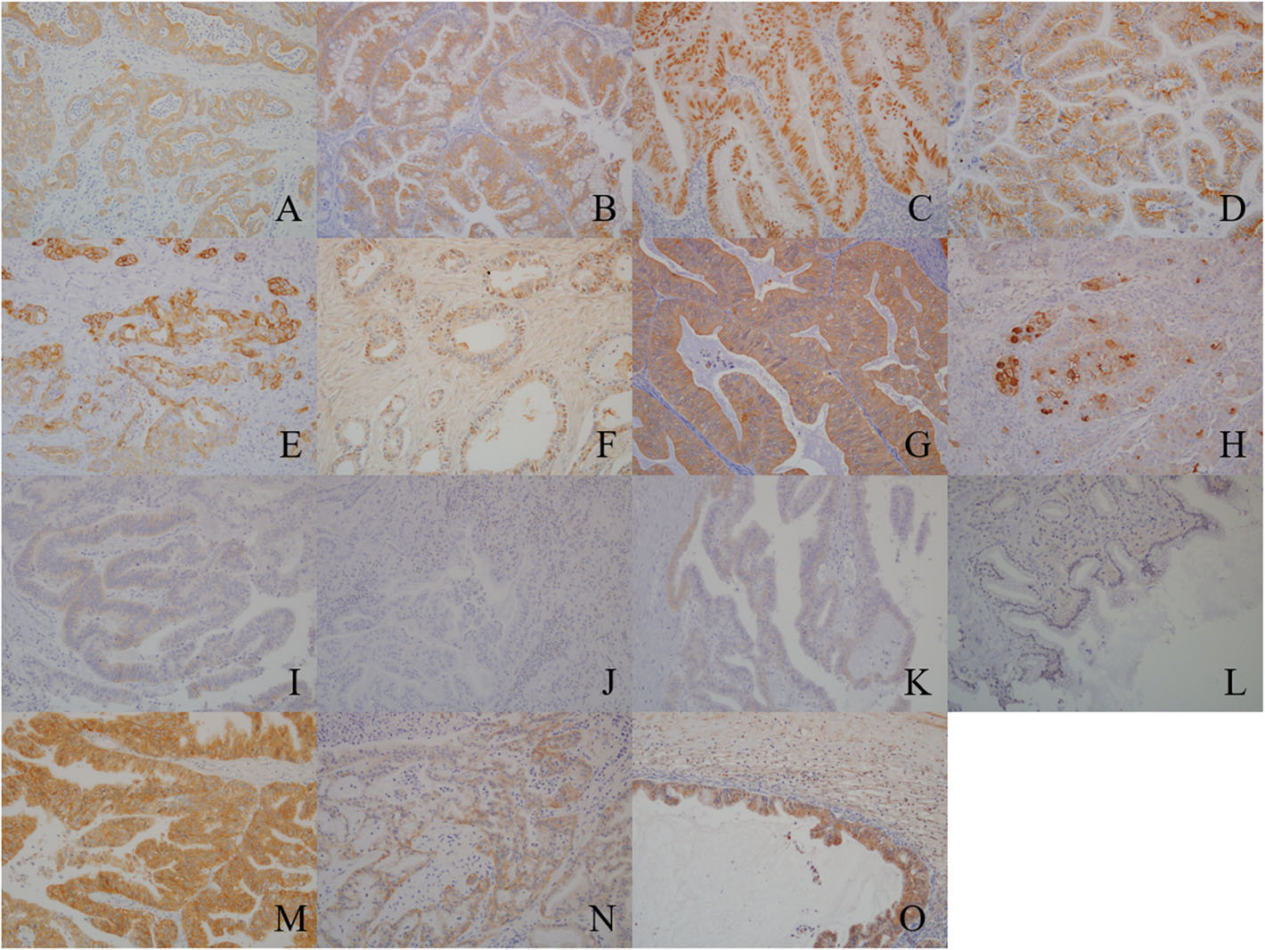
Representative images of positive cells of each immunohistochemistry **a** Cytokeratin 7 (x200); **b** Cytokeratin 20 (x200); **c** Caudal type homeobox 2 (x200); **d** Human epidermal growth factor receptor 2 (x200); **e** Cytokeratin 5/6 (x200); **f** Androgen receptor (x200); **g** Aldehyde dehydrogenase 1 (x200); **h** Cluster of differentiation 24 (x200); **i** Cluster of differentiation 133 (x200); **j** Programmed cell death 1 (x200); **k** Programmed cell death 1- ligand 1 (x200); **l** Zinc finger E-box binding homeobox 1 (x200); **m** Met (x200); **n **Epithelial growth factor receptor (x200); **o** Snail-2 (x200)

Formalin-fixed paraffin-embedded tissues were used for immunohistochemistry. All the slides were deparaffinized and rehydrated using graded ethanol series. The endogenous peroxidase activity was blocked by adding methanol to 0.3 % hydrogen peroxidase. Antigen retrieval was performed using citrate buffer (pH 6.0) and Tris/EDTA buffer (pH 9.0). Additionally, all the slides were boiled in an autoclave at 121°C for 5 min in a citrate buffer or in Tris/EDTA buffer at 98°C for 40 min. The primary antibodies are listed in Table 5. All the slides were incubated with these antibodies at room temperature for 24 h. Following incubation, the samples were reacted with the DAKO EnVision + system-HRP labeled polymer as a secondary antibody for 30 min at room temperature. Specific antigen-antibody reactions were visualized with 0.2 % diaminobenzidine tetrahydrochloride and hydrogen peroxide and counterstained with Mayer’s hematoxylin. The control tissue sections and interpretations for each antibody are listed in Table 5. In case of the presence of tumor cells, the representative images of positive cells from each immunohistochemical staining are shown in Fig. 5.

**Table 5:** Primary antibodies

Molecule	Type	Antibody Clone/Code	Manufacturer	Dilution	Localization	Control tissue	Antigen retrieval	Interpretation
CK7	Monoclonal (Mouse)	OV-TL12/30	Dako	x50	Cytoplasm	Colorectal carcinoma	Citrate	More than 10 % positive cell with staining intensity score 1 to 3 was defined as positive.
CK20	Monoclonal (Mouse)	Ks20.8	Dako	x50	Cytoplasm	Colorectal carcinoma	Citrate	More than 10 % positive cell with staining intensity score 1 to 3 was defined as positive.
CDX2	Monoclonal (Rabbit)	CDX2-88	Novocastra	x100	Nucleus	Colon	Citrate	More than 10 % positive cell with staining intensity score 1 to 3 was defined as positive.
HER-2	Monoclonal (Rabbit)	4B5	Ventana medical system	Ready to use	Membrane	Mammary gland	Citrate	More than 10 % positive cell with staining intensity score 1 to 3 was defined as positive.
CK 5/6	Monoclonal (Mouse)	D5/13 B4	Dako	x100	Cytoplasm	Mammary gland	EDTA	More than 10 % positive cell with staining intensity score 1 to 3 was defined as positive.
Androgen receptor	Monoclonal (Mouse)	AR441	Dako	x50	Nucleus	Mammary gland	Citrate	More than 10 % positive cell with staining intensity score 1 to 3 was defined as positive.
ALDH1	Monoclonal (Mouse)	clone 44	BD Biosciences	x500	Cytoplasm	Kidney	Citrate	More than 10 % positive cell with staining intensity score 1 to 3 was defined as positive.
CD24	Monoclonal (Mouse)	SN3	ThermoFisher	x25	Membrane	Tonsil	Citrate	More than 10 % positive cell with staining intensity score 1 to 3 was defined as positive.
CD133	Monoclonal (Mouse)	AC133/1	Miltenyi Biotec	x500	Cytoplasm	Colorectal carcinoma	EDTA	More than 10 % positive cell with staining intensity score 1 to 3 was defined as positive.
PD-1	Monoclonal (Mouse)	NAT105	Abcam	x50	lymphocytes	Tonsil	Citrate	Even minimally positive lymphocyte with staining intensity score 1 to 3 was defined as positive.
PD-L1	Monoclonal (Rabbit)	EPR19759	Abcam	x250	Membrane and endomembrane	Placenta	Citrate	More than 10 % positive cell with staining intensity score 1 to 3 was defined as positive.
ZEB1	Polyclonal (Rabbit)	ab87280	Abcam	x100	Nucleus	MCF, MDA-MB-231	EDTA	More than 1 % positive cell with staining intensity score 1 to 3 was defined as positive.
c-Met	Monoclonal (Rabbit)	D1C2	Cell Signaling Technology	x300	Membrane and cytoplasm	Colorectal carcinoma	Citrate	More than 10 % positive cell with staining intensity score 1 to 3 was defined as positive.
EGFR	Monoclonal (Rabbit)	D38B1	Cell Signaling Technology	x100	Membrane	Epidermis	EDTA	More than 10 % positive cell with staining intensity score 2 or 3 was defined as positive.
Snail-2	Polyclonal (Rabbit)	PAB1923	Abnova	x200	Nucleus	MCF, MDA-MB-231	Citrate	More than 10 % positive cell with staining intensity score 1 or 3 was defined as positive.

Abbreviation: *CK7* Cytokeratin 7, *CK20* Cytokeratin 20, *CDX2* Caudal type homeobox 2, *HER-2* Human epidermal growth factor receptor 2, *CK5/6* Cytokeratin 5/6, *ALDH1* Aldehyde dehydrogenase 1, *CD24* Cluster of differentiation 24, *CD133* Cluster of differentiation 133, *PD-1* Programmed cell death 1, *PD-L1* Programmed cell death 1- ligand 1, *ZEB1* Zinc finger E-box binding homeobox 1, *EGFR* Epithelial growth factor receptor

Proportion score, defined as the percentage of cells in carcinoma tissues, was as follows:0, no tumor cells stained in entire carcinoma tissue; 1+, form ≥ 1 % to < 10 % cells stained in entire carcinoma tissue; 2+, from ≥ 10 % to < 50 %; 3+, ≥ 50 %. Staining intensity score is defined as follows:0, no tumor cells stained in entire carcinoma tissue; 1+, incomplete staining and/or faint or barely perceptible cytoplasmic staining detected; 2+, entire carcinoma cells stained and/or moderate to strong staining. Immunochemical interpretation was listed Table 5.

According to the protocol at our hospital, all the patients preoperatively underwent gastroscopy and colonofiberscopy to exclude the metastatic OC originating from the gastrointestinal tract. Furthermore, when swelling of the appendix was observed intraoperatively, resection of the appendix was performed by a digestive surgeon to exclude appendiceal cancer.

Clinical information was obtained from the medical records. The tumor stage was reevaluated according to the 2014 FIGO criteria [3]. The standard surgery was bilateral salpingo-oophorectomy with hysterectomy, omentectomy, or multiple peritoneal biopsy and lymphadenectomy. The residual tumor was evaluated using the operation records after the primary surgery. The Response Evaluation Criteria in Solid Tumors version 1.1 [39] were used to evaluate the treatment effectiveness. Progression-free survival (PFS) was defined as the period from the day of PDS to the day of death or recurrence/progression of the disease. Overall survival (OS) was defined as the period from the day of PDS to the day of death or the last confirmation of survival.

Statistical analysis was performed using JMP Pro 14 software (SAS Institute Inc., Cary, NC, USA). The chi-square test, Fisher’s exact test, Mann-Whitney U test, and Wilcoxon test were used to evaluate the clinical significance of the clinicopathological factors. The PFS and OS curves were generated using the Kaplan-Meier method. The survival distribution was compared using the log-rank test. Univariate and multivariate analysis was performed by cox proportional hazard regression models. Firstly, Univariate analysis for PFS and OS was performed with several factors. Secondly, multivariate analysis for PFS and OS was done using the only factors with statistical significances by univariate analysis as the parameter. Statistical significance was defined as *p* < 0.05. This study was approved by the Ethics Committee of the National Defense Medical College, Tokorozawa, Japan.

## Data Availability

The dataset supporting the conclusions of this article is included within the article.

## Abbreviations

OC: Ovarian carcinoma
FIGO: The International Federation of Gynecology and Obstetrics
OMC: Ovarian mucinous carcinoma
CK: Cytokeratin
CD: Cluster of differentiation
EGFR: Epithelial growth factor receptor
PFS: Progression free survival
PDS: Primary debulking surgery
OS: Overall survival
CK: Cytokeratin
CD: Cluster of differentiation
EGFR: Epithelial growth factor receptor
